# The Utilization of Systematic Reviews and Meta-Analyses in Stroke Guidelines

**DOI:** 10.3390/brainsci14070728

**Published:** 2024-07-20

**Authors:** Sherief Ghozy, Hassan Kobeissi, Melika Amoukhteh, Ramanathan Kadirvel, Waleed Brinjikji, Alejandro A. Rabinstein, Christopher R. Carpenter, David F. Kallmes

**Affiliations:** 1 Department of Radiology, Mayo Clinic, Rochester, MN 55905, USA; kobei1h@cmich.edu (H.K.); dr.amoukhteh@gmail.com (M.A.); kadir@mayo.edu (R.K.); brinjikji.waleed@mayo.edu (W.B.); kallmes.david@mayo.edu (D.F.K.); 2 Department of Neurologic Surgery, Mayo Clinic, Rochester, MN 55905, USA; 3 Department of Neurology, Mayo Clinic, Rochester, MN 55905, USA; rabinstein.alejandro@mayo.edu; 4 Department of Emergency Medicine, Mayo Clinic, Rochester, MN 55905, USA; carpenter.christopher@mayo.edu

**Keywords:** stroke, guidelines, systematic review, meta-analysis

## Abstract

Background: Stroke guideline statements are important references for clinicians due to the rapidly evolving nature of treatments. Guideline statements should be informed by up-to-date systematic reviews (SRs) and meta-analyses (MAs) because they provide the highest level of evidence. To investigate the utilization of SRs/MAs in stroke management guidelines, we conducted a literature review of guidelines and extracted relevant information regarding SRs/MAs. Methods: A literature review was conducted in PubMed with supplementation using the Trip medical database with the term “stroke” as the target population, followed by using the filter “guidelines”. We extracted the number of included SRs/MAs, the years of publication, the country of origin, and other characteristics of interest. Descriptive statistics were generated using the R software version 4.2.1. Results: We included 27 guideline statements. The median number of overall SRs or MAs within the guidelines was 4.0 (interquartile range [IQR] = 2–9). For MAs only, the median number included in the guidelines was 3.0 (IQR = 2.0–5.5). Canadian guidelines had the oldest citations, with a median gap of 12.0 (IQR = 5.2–18.0) years for the oldest citation, followed by European (median = 12; IQR = 9.5–13.5) and US (median = 10.0; IQR = 5.2–16) guidelines. Conclusions: Stroke guideline writing groups and issuing bodies should devote greater effort to the inclusion of up-to-date SRs/MAs in their guideline statements so that clinicians can reference recent data with the highest level of evidence.

## 1. Introduction

The management of stroke, and particularly acute ischemic stroke (AIS), is rapidly evolving. New treatment modalities, indications, and management strategies have emerged following the proven superiority of endovascular therapy (EVT) over medical management for AIS due to large vessel occlusion (LVO) [1]. Furthermore, the use of medical therapies and intravenous thrombolysis is also rapidly evolving, with the use of neuroprotective agents and different thrombolytic drugs being continuously investigated [2,3,4].

Due to the rapidly evolving nature of stroke management, guideline statements are necessary to improve patient care and to ensure that clinicians are up to date on the latest evidence. For example, one academic emergency department reported that the implementation of guidelines decreased the time taken to conduct the assessment and treatment of stroke patients [5]. According to the Institute of Medicine (IOM) and the Grading of Recommendations Assessment, Development, and Evaluation (GRADE) system, the foundation of guideline statements should be systematic reviews (SRs) [6,7]. SRs and meta-analyses (MAs) offer the highest level of evidence, often with large sample sizes and the representation of multi-center results [8]. The American Heart Association (AHA) considers evidence derived from MAs to have a level “A” rating—in other words, the highest possible rating.

Despite the clear benefits of SRs/MAs, their incorporation into guideline statements may be underutilized. Moreover, even when SRs/MAs are included in guideline statements, their recency and relevancy may not always reflect the latest evidence.

To investigate the quantity and recency of SRs/MAs in stroke management guidelines, we conducted a literature review of guidelines and extracted relevant information regarding SRs/MAs.

## 2. Methods

### 2.1. Literature Search, Screening, and Data Extraction

On 10 November 2023, a literature review was conducted in PubMed using the terms “stroke” and “guidelines”, with a limitation to the presence of these terms in the title of the paper to exclude irrelevant studies. Because not all guidelines are PubMed-indexed, we supplemented the search with the Trip medical database (https://www.tripdatabase.com accessed on 10 November 2023) with the term “stroke” as the target population, followed by the filter “guidelines”. No restrictions were placed on the date of publication.

Two authors exported and screened the results for possible inclusion, and a third senior author resolved any inconsistencies in screening through discourse and a thorough review of the extracted data. We defined a guideline as any work self-identified by the authors as a “guideline” and issued by a national research group or society, while excluding guidelines written in a non-English language, policy papers, single-institution ones, or those with unknown issuing entities. In the case of multiple versions of a guideline, we included only the most recent one. Living practice guidelines are “an optimization of the guideline development process to allow updating of individual recommendations as soon as relevant new evidence becomes available” [9,10]. There was only one living guideline (eight chapters), produced by the Stroke Foundation (Australia and New Zealand) [11], so we excluded it to maintain consistency and avoid problems associated with the continuous citation update. At least two authors extracted all relevant data from the included guidelines, and a senior author confirmed the accuracy of all data and resolved any discrepancies.

### 2.2. Statistical Analysis

Descriptive statistics were generated using the R software version 4.2.1 [12], using the (Rcmdr) [13] package. Categorical data were represented as counts and frequencies, while medians and interquartile ranges (IQR) were used for skewed continuous variables. The skewness and kurtosis tests were used to assess the normal distribution of continuous variables.

## 3. Results

### 3.1. Summary of the Included Guidelines

The search strategy retrieved 245 results in PubMed and 142 from the Trip database. Following the exclusion of duplicate records, older versions, and irrelevant results, we ultimately included 27 guidelines (Table S1 in the Supplementary Materials). Regarding the source country, US entities were the sources of 37.0% of the included sample, followed by Canada (18.5%), China (14.8%), and Europe (11.1%). Over half of the included sample was issued between 2019 and 2020 (59.2%), with the AHA (22.2%), the Chinese Stroke Association (CSA) (14.8%), and Thrombosis Canada (11.1%) as the top three issuing bodies in terms of the quantity of guidelines (Table A1).

### 3.2. Systematic Reviews’ Utilization

**Figure 1:**
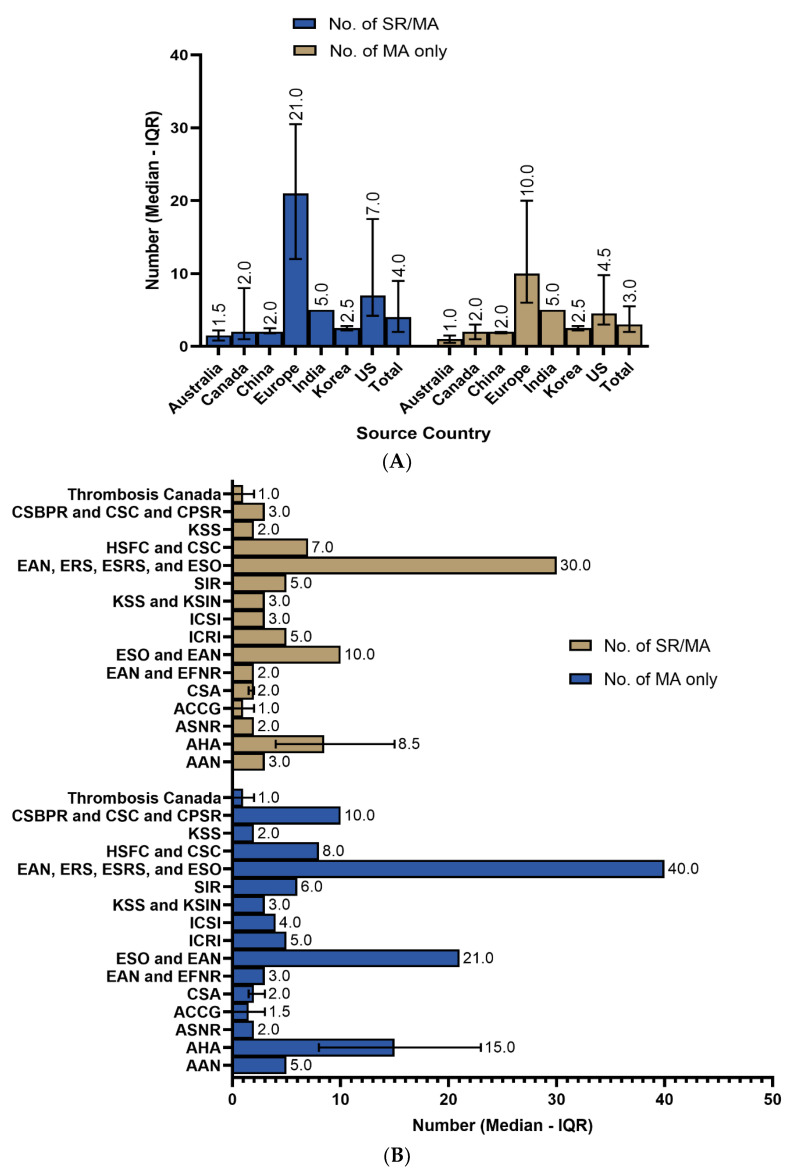

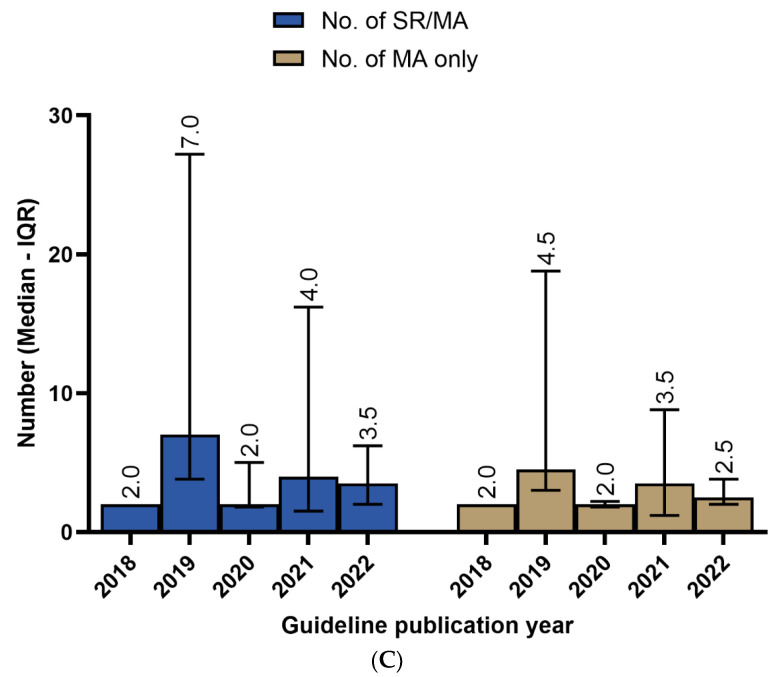
(**A**) Number of systematic reviews and/or meta-analyses in stroke guidelines sorted by source country. (**B**) Number of systematic reviews and/or meta-analyses in stroke guidelines sorted by issuing body and (**C**) sorted by guideline’s publication year. AAN: American Academy of Neurology; AHA: American Heart Association; ASNR: American Society of Neuroradiology; ACCG: Australian Clinical Consensus Guideline; CSA: Chinese Stroke Association; EAN: European Academy of Neurology; EFNR: European Federation of Neurorehabilitation; ESO: European Stroke Organisation; ICRI: Indian College of Radiology and Imaging; ICSI: Institute for Clinical Systems Improvement; KSS: Korean Stroke Society; KSIN: Korean Society of Interventional Neuroradiology; SIR: Society of Interventional Radiology; CSBPR: Canadian Stroke Best Practice Recommendations; CSC: Canadian Stroke Consortium; CPSR: Canadian Partnership for Stroke Recovery; ERS: European Respiratory Society; ESRS: European Sleep Research Society; HSFC: Heart and Stroke Foundation of Canada.

The median number of the overall SRs or MAs within the guidelines was 4.0 (IQR = 2.0–10.0). For meta-analyses only, the median number included in the guidelines was 3.0 (IQR = 2.0–6.0). The median gap between the year of issuing the guideline and the cited SRs/MAs was as high as 8.0 (IQR = 5.0–15.0) years and as low as 2.0 (IQR = 1.0–3.0) years (Table A2). On further exploration of the results, the European guidelines had the highest utilization of SRs/MAs, with a median of 21 (IQR = 12–30.5) citations, followed by US guidelines with 7.0 (IQR = 4.2–17.5) citations. Regarding MAs only, the median citations were 10.0 (IQR= 6.0–20.0) for Europe and 4.5 (IQR = 3.0–9.8) for the US (Figure 1A). Similar trends were observed regarding the issuing bodies, with European organizations having the highest number of citations, followed by American organizations (Figure 1B). Guidelines released in 2019 had the highest utilization of SRs/MAs (median = 7.0; IQR = 3.8–27.2), followed by 2021 (median = 4.0; IQR = 1.5–16.2) and 2022 (median = 3.5; IQR = 2.0–6.2) (Figure 1C).

### 3.3. Recency of SRs/MAs

**Figure 2:**
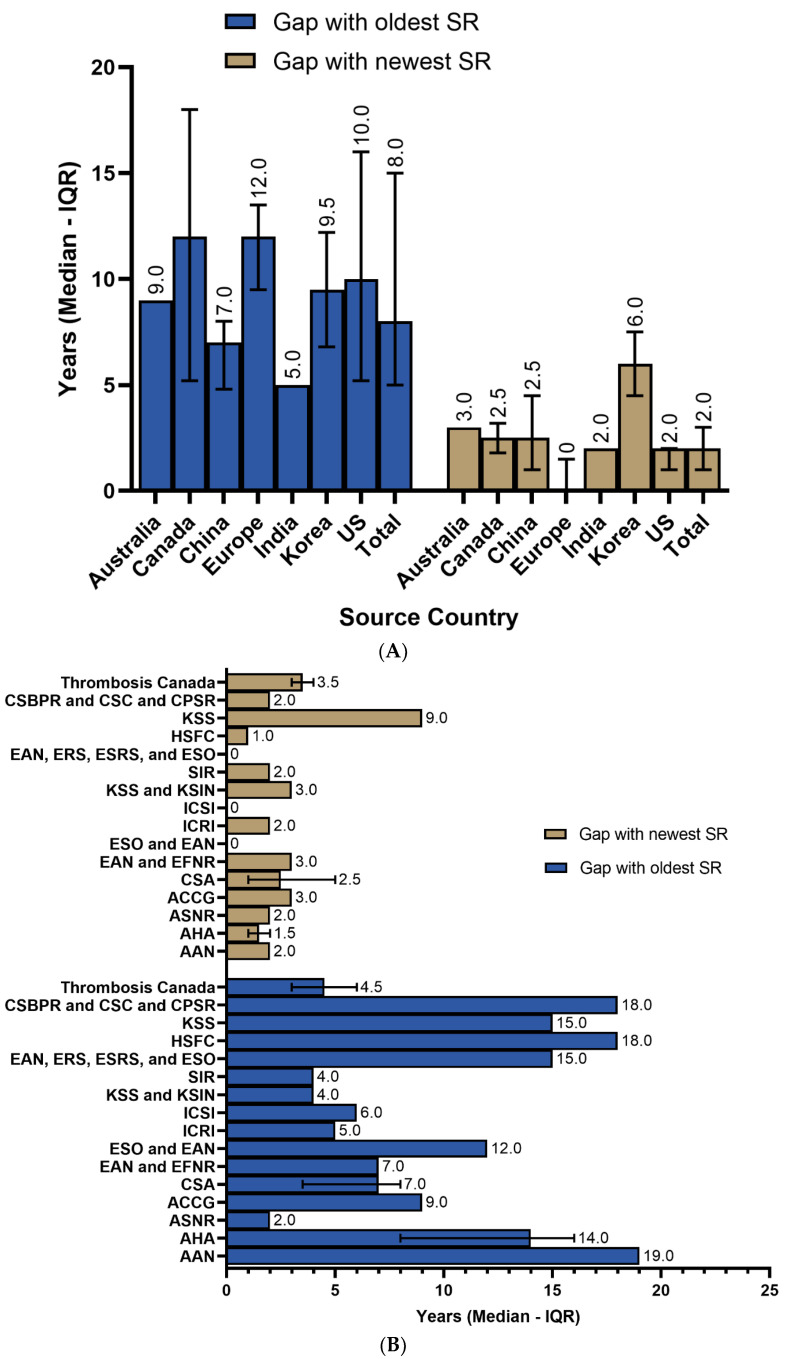

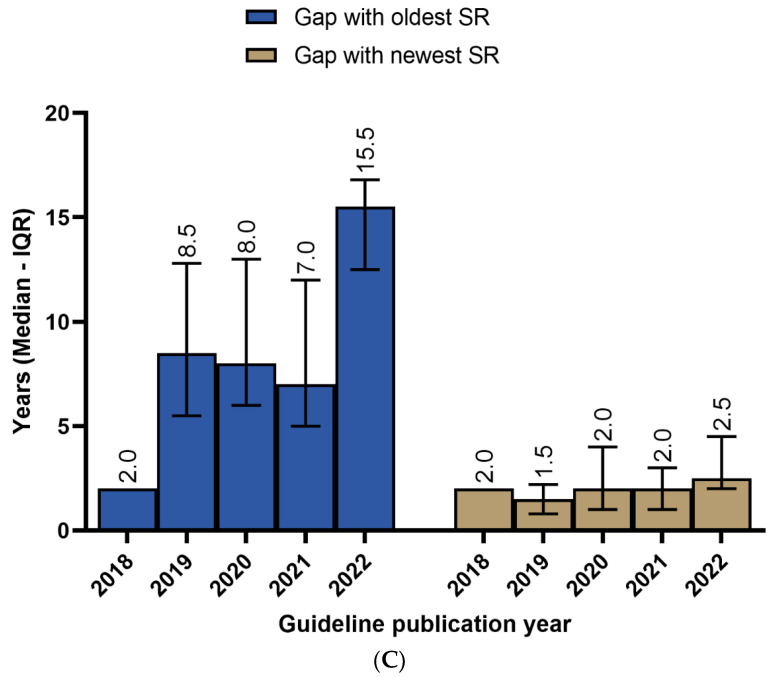
(**A**) Gap years from the publication year of stroke guidelines sorted by source country. (**B**) Gap years from the publication year of stroke guidelines sorted by issuing body. AAN: American Academy of Neurology; AHA: American Heart Association; ASNR: American Society of Neuroradiology; ACCG: Australian Clinical Consensus Guideline; CSA: Chinese Stroke Association; EAN: European Academy of Neurology; EFNR: European Federation of Neurorehabilitation; ESO: European Stroke Organisation; ICRI: Indian College of Radiology and Imaging; ICSI: Institute for Clinical Systems Improvement; KSS: Korean Stroke Society; KSIN: Korean Society of Interventional Neuroradiology; SIR: Society of Interventional Radiology; CSBPR: Canadian Stroke Best Practice Recommendations; CSC: Canadian Stroke Consortium; CPSR: Canadian Partnership for Stroke Recovery; ERS: European Respiratory Society; ESRS: European Sleep Research Society; HSFC: Heart and Stroke Foundation of Canada. (**C**) Gap years from the publication year of stroke guidelines sorted by guideline’s publication year.

To investigate how up to date the cited SRs/MAs were, we calculated the gap between the year of the guideline and the SR/MA’s publication year. The overall median number of gap years with the oldest SR/MA was 8.0 (IQR = 5.0–15.0), while it was 2.0 (IQR = 1.0–3.0) from the newest one. Canadian guidelines had the oldest citations, with a median gap of 12.0 (IQR = 5.2–18.0) years for the oldest citation, followed by European (median = 12; IQR = 9.5–13.5) and US guidelines (median = 10.0; IQR = 5.2–16) guidelines. European, US, and Indian guidelines had 0 (IQR = 0–1.5), 2.0 (IQR = 1.0–2.0), and 2 (IQR = 2.0–2.0) year gaps from the newest cited SR/MA, respectively (Figure 2A). On sorting the same data based on the issuing body, the American Academy of Neurology (ANA) had the oldest included citations, followed by the Heart and Stroke Foundation of Canada (HSFC) and Canadian Stroke Consortium (Figure 2B). Moreover, the European guidelines and the Institute for Clinical Systems Improvement (ICSI) included the most recent systematic reviews with no gap years (Figure 2B). The guidelines issued in 2022 had the widest gap years, with the oldest included SRs/MAs (median = 15.5; IQR = 12.5–16.8), followed by 2019 (median = 8.5; IQR = 5.5–12.8) and 2020 (median = 8.0; IQR = 6.0–13.0) guidelines. In addition, the most recent SRs/MAs were included in the guidelines released in 2019 (median gap years = 1.5; IQR = 0.8–2.2), while more recent years had a comparable median gap of two or more years with the newest included SR/MA (Figure 2C).

## 4. Discussion

In this study, we found that SRs/MAs were underutilized in stroke guidelines and that the SRs/MAs included in the guidelines tended to be multiple years old. Our results suggest that there are opportunities to improve the incorporation of SRs/MAs in stroke management guidelines. Furthermore, due to the rapidly evolving nature of stroke treatments, an effort should be made to ensure that the most up-to-date SRs/MAs are incorporated into the guidelines. Our findings also indicate that the utilization rate of SRs/MAs is not increasing. We found that the most recent guidelines did not have the lowest number of gap years regarding SRs/MAs. This observation aligns with findings from other medical fields. For instance, a study on the uptake of individual participant data (IPD) MAs in clinical practice guidelines found that only 37% of guidelines cited a relevant IPD meta-analysis, and 27% clearly used this information in formulating recommendations [14]. Guerra-Farfan et al. noted that clinical practice guidelines often suffer from biases, limitations, and outdated recommendations due to the lengthy development process and conflicts of interest among panel members [15]. This suggests a broader issue across various medical disciplines, where the most robust evidence is underutilized in clinical guidelines. While important for all guideline statements, the utilization of SRs/MAs is especially pertinent for stroke guidelines. For example, two new trials published in 2022 showed the superiority of EVT over medical therapy for posterior circulation AIS in eligible patients [16,17]. Following this, a recent, higher-powered MA was able to prove the superiority of EVT for posterior circulation AIS due to LVO using data from all available randomized controlled trials [18]. Prior to these results, the superiority of EVT was not definitive in this patient population, and new guidelines will likely incorporate these findings. This is one of many examples of the rapidly evolving nature of stroke management.

The number of SRs being published has been steadily increasing. A recent study found that, in 2019, there were around 80 new SRs indexed on PubMed per day [19]. In comparison, there were four new SRs indexed on PubMed per day in 2000, indicating that there has been a 20-fold increase in the number of SRs being published over the last two decades [19]. Similar trends were present for MAs, with 1000 total PubMed-indexed MAs in the year 2000, compared to over 11,000 in 2017. This trend can be attributed to the fact that SRs/MAs are increasingly recognized as the highest level of evidence and that they can be conducted with relatively few resources compared to clinical trials. Although the number of published SRs/MAs is increasing, we did not find a similar trend in the utilization of SRs/MAs in the development of practice guidelines. The aforementioned statistics indicate that the lack of utilization of SRs/MAs cannot be attributed to a lack of literature, as there has been a rapid increase in the number of SRs/MAs.

The low utilization of SRs/MAs in stroke guidelines could be attributed to several critical factors. Firstly, the field of stroke management is rapidly evolving, with new treatments and management strategies continuously emerging [20,21]. This dynamic nature means that the guidelines often lag behind the latest evidence. For instance, advancements in endovascular therapy and novel thrombolytic agents are frequent, and, by the time that the guidelines are published, some included SRs and MAs may already be outdated. Secondly, the process of developing and updating guidelines is inherently time-consuming and involves extensive review and consensus building [15,22,23]. This can result in a significant time gap between the publication of new evidence and its incorporation into the guidelines. Additionally, the variability in resources and priorities among different countries and organizations can impact the frequency and comprehensiveness of guideline updates. Some regions may lack the necessary infrastructure or funding to perform regular and thorough updates, or they simply do not prioritize the recency of the evidence, leading to reliance on older evidence. This trend is similar to what was observed in a previous study, which highlighted that guideline development practices vary significantly across regions, affecting the quality and recency of the guidelines [24]. It was also noted that over half of the guidelines used non-systematic methods to synthesize the evidence to inform recommendations [25]. Furthermore, guideline panels may prioritize recently published primary studies, especially RCTs, over SRs/MAs due to the immediacy and relevance of new primary data. However, using individual RCTs without appropriate quality assessments and weighing of the risk of bias, as is seen in SRs/MAs, could be inappropriate in terms of the possible flaws of some RCTs [26,27,28]. Lastly, methodological challenges in incorporating SRs/MAs into guidelines cannot be overlooked. Discrepancies between different SRs/MAs, the varying quality of the included studies, and differences in methodological approaches can complicate their integration into cohesive guideline statements [28]. In this context, a possible reason may be the lack of trust for non-Cochrane reviews; between 1993 and 2002, Cochrane reviews represented 35% of all systematic reviews indexed in PubMed, compared to just 3.5% in the period from 2013 to 2022. The highest-quality SRs/MAs should adhere to the appropriate reporting guidelines and pool evidence from randomized controlled trials [29]. Furthermore, the protocols of SRs/MAs should be prospectively registered in databases to avoid the duplication of studies and to increase the transparency [30]. Further research is needed to investigate the quality of the SRs/MAs included in stroke guidelines. However, this may not be plausible justification to depend on a small portion of evidence from a single source.

Our study suffered from limitations. Since there was no provided justification by the authors of the guidelines for the inclusion/exclusion of specific SRs/MAs, we could not account for some factors, such as the methodological quality or the added value of updated studies on the same topic. Moreover, older studies may have been used in a necessary context; again, no systematic approach or justification was provided. A deeper analysis that incorporates the authors’ input is needed for more concrete conclusions. This should take into account why the authors decided to include specific SRs/MAs, how their research question may/may not have been affected by their recency, how many authors performed their own reviews and how, the quality of the included studies, and the level of quality that is considered sufficiently high by the authors to be included.

To address the gap in utilizing MAs in clinical practice guidelines, several key strategies are recommended. Guideline writing groups should establish formal processes for regular updates to incorporate the latest SRs and MAs, as emphasized by others, to ensure that the guidelines reflect the current evidence [14]. Addressing conflicts of interest through stringent policies and transparency can reduce biases [15]. Enhanced stakeholder involvement, including patients and clinicians, can ensure comprehensive and balanced recommendations, addressing challenges in incorporating patient values [15]. Utilizing systematic and transparent evidence synthesis methods can improve the reliability of guidelines [25]. Providing training for guideline developers on advanced evidence synthesis techniques can build capacity and bridge gaps in utilizing MAs. Additionally, implementing living guidelines, continuously updated with new evidence, can prevent outdated recommendations and ensure the real-time incorporation of research findings.

## 5. Conclusions

In this study, we found that SRs/MAs were variably and insufficiently utilized in stroke guidelines and that they were often out of date. Guideline writing groups and issuing bodies should seek to increase the utilization of up-to-date SRs/MAs due to the rapidly evolving nature of evidence in regard to stroke management.

## Data Availability

The original contributions presented in the study are included in the article/Supplementary Material; further inquiries can be directed to the corresponding author.

## Supplementary Materials

The following supporting information can be downloaded at: https://www.mdpi.com/article/10.3390/brainsci14070728/s1, Table S1: List of the included guidelines.

## Appendix A

**Table A1:** Summary of the included guidelines.

Source Country	Overall (N = 27)
Australia	2 (7.4%)
Canada	5 (19%)
China	4 (15%)
Europe	3 (11%)
India	1 (3.7%)
Korea	2 (7.4%)
US	10 (37%)
**Guideline Publication Year**	
2018	1 (3.7%)
2019	8 (30%)
2020	8 (30%)
2021	6 (22%)
2022	4 (15%)
**Issuing Body**	
AAN	1 (3.7%)
AHA	6 (22%)
ASNR	1 (3.7%)
ACCG	2 (7.4%)
CSA	4 (15%)
EAN and EFNR	1 (3.7%)
ESO and EAN	1 (3.7%)
ICRI	1 (3.7%)
ICSI	1 (3.7%)
KSS and KSIN	1 (3.7%)
SIR	1 (3.7%)
CSBPR, CSC, and CPSR	1 (3.7%)
Thrombosis Canada	3 (11%)
EAN, ERS, ESRS, and ESO	1 (3.7%)
HSFC and CSC	1 (3.7%)
KSS	1 (3.7%)

AAN: American Academy of Neurology; AHA: American Heart Association; ASNR: American Society of Neuroradiology; ACCG: Australian Clinical Consensus Guideline; CSA: Chinese Stroke Association; EAN: European Academy of Neurology; EFNR: European Federation of Neurorehabilitation; ESO: European Stroke Organisation; ICRI: Indian College of Radiology and Imaging; ICSI: Institute for Clinical Systems Improvement; KSS: Korean Stroke Society; KSIN: Korean Society of Interventional Neuroradiology; SIR: Society of Interventional Radiology; CSBPR: Canadian Stroke Best Practice Recommendations; CSC: Canadian Stroke Consortium; CPSR: Canadian Partnership for Stroke Recovery; ERS: European Respiratory Society; ESRS: European Sleep Research Society; HSFC: Heart and Stroke Foundation of Canada.

**Table A2:** Summary of systematic reviews’ utilization in stroke guidelines.

Variable	Median	IQR
Number of systematic reviews and/or meta-analyses	4.0	2–10
Number of meta-analyses only	3.0	2–6
Gap with oldest (years)	8.0	5–15
Gap with newest (years)	2.0	1–3

IQR: interquartile range.
