# Supplementary material for: The Utilization of Systematic Reviews and Meta-Analyses in Stroke Guidelines
Source: Brain Sci. 2024 Jul 20;14(7):728. doi: 10.3390/brainsci14070728 (PMC11274449; doi:10.3390/brainsci14070728)
Supplement: Supplementary file 1 [file brainsci-14-00728-s001.zip › brainsci-3040525-supplementary.pdf]

Table S1. List of the included guidelines.

Title	Source Country	Main issuing body	Guideline publication year	Link
Australian clinical consensus guideline for the subacute rehabilitation of childhood stroke	Australia	Australian Clinical Guidelines	2021	https://pubmed.ncbi.nlm.nih.gov/32691701/
Australian Clinical Consensus Guideline: The diagnosis and acute management of childhood stroke	Australia	Australian Clinical Guidelines	2019	https://pubmed.ncbi.nlm.nih.gov/30284961/
Canadian Stroke Best Practice recommendations, seventh edition: acetylsalicylic acid for prevention of vascular events	Canada	the Heart and Stroke Foundation of Canada in collaboration with the Canadian Stroke Consortium	2020	https://www.cmaj.ca/content/cmaj/192/12/E302.full.pdf
Canadian Stroke Best Practice recommendations: rehabilitation, recovery, and community participation following stroke. Part one: rehabilitation and recovery following stroke; 6th edition update 2019	Canada	The Management of Rehabilitation and Recovery following Stroke Best Practice Writing Group and the Canadian Stroke Best Practices and Quality Advisory Committee, and in collaboration with the Canadian Stroke Consortium and the Heart & Stroke Canadian Partnership for Stroke Recovery	2020	https://journals.sagepub.com/doi/pdf/10.1177/1747493019897843
Ischemic Stroke or TIA: Secondary Prevention	Canada	Thrombosis Interest Group of Canada	2021	https://thrombosiscanada.ca/clinicalguides/
Stroke Prevention in Atrial Fibrillation	Canada	Thrombosis Interest Group of Canada	2020	https://thrombosiscanada.ca/clinicalguides/
Stroke: Thrombolysis and Endovascular Therapy	Canada	Thrombosis Interest Group of Canada	2020	https://thrombosiscanada.ca/clinicalguides/
Chinese Stroke Association guidelines for clinical management of cerebrovascular disorders: executive summary and 2019 update of clinical management of ischaemic cerebrovascular diseases	China	Chinese Stroke Association Stroke Council Guideline Writing Committee	2020	https://pubmed.ncbi.nlm.nih.gov/32561535/

Chinese Stroke Association guidelines for clinical management of cerebrovascular disorders: executive summary and 2019 update of clinical management of stroke rehabilitation	China	Chinese Stroke Association Stroke Council Guideline Writing Committee	2020	https://pubmed.ncbi.nlm.nih.gov/32595138/
Chinese Stroke Association guidelines for clinical management of cerebrovascular disorders: executive summary and 2019 update of the management of high-risk population	China	Chinese Stroke Association Stroke Council Guideline Writing Committee	2020	https://pubmed.ncbi.nlm.nih.gov/32792457/
Chinese Stroke Association guidelines for clinical management of cerebrovascular disorders: executive summary and 2019 update on organizational stroke management	China	Chinese Stroke Association Stroke Council Guideline Writing Committee	2020	https://pubmed.ncbi.nlm.nih.gov/32641444/
EAN/ERS/ESO/ESRS statement on the impact of sleep disorders on risk and outcome of stroke	Europe	the European Academy of Neurology (EAN), the European Respiratory Society (ERS), the European Sleep Research Society (ESRS), and the European Stroke Organization (ESO)	2019	https://erj.ersjournals.com/content/55/4/1901104
European Academy of Neurology and European Federation of Neurorehabilitation Societies guideline on pharmacological support in early motor rehabilitation after acute ischaemic stroke	Europe	European Academy of Neurology and European Federation of Neurorehabilitation Societies	2021	https://onlinelibrary.wiley.com/doi/10.1111/ene.14936
European Stroke Organisation and European Academy of Neurology joint guidelines on post-stroke cognitive impairment	Europe	European Stroke Organisation and European Academy of Neurology	2021	https://onlinelibrary.wiley.com/doi/10.1111/ene.15068
Indian College of Radiology and Imaging (ICRI) Consensus Guidelines for the Early Management of Patients with Acute Ischemic Stroke: Imaging and Intervention	India	Indian College of Radiology and Imaging (ICRI)	2021	https://pubmed.ncbi.nlm.nih.gov/34556925/
2019 Update of the Korean Clinical Practice Guidelines of Stroke for Endovascular Recanalization Therapy in Patients with Acute Ischemic Stroke	Korea	Korean Stroke Society and the Korean Society of Interventional Neuroradiology	2019	https://pubmed.ncbi.nlm.nih.gov/30991800/
2022 Update of the Korean Clinical Practice Guidelines for Stroke: Antithrombotic Therapy for Patients with Acute Ischemic Stroke or Transient Ischemic Attack	Korea	the Korean Stroke Society	2022	https://pubmed.ncbi.nlm.nih.gov/35135073/

ACR–ASNR–SIR–SNIS Practice Parameter for the Performance of Endovascular Embolectomy and Revascularization in Acute Stroke	US	American Society of Neuroradiology	2018	https://www.asnr.org/wp-content/uploads/2019/06/Acute-Stroke-1.pdf
Guidelines for the Early Management of Patients With Acute Ischemic Stroke: 2019 Update to the 2018 Guidelines for the Early Management of Acute Ischemic Stroke: A Guideline for Healthcare Professionals From the American Heart Association/American Stroke Association	US	American Heart Association	2019	https://www.ahajournals.org/doi/10.1161/STR.0000000000000211
Health Care Guideline: Diagnosis and Initial Treatment of Ischemic Stroke	US	Institute for Clinical Systems Improvement	2019	https://www.icsi.org/wp-content/uploads/2019/07/ICSI_Stroke_June2019_v2.pdf
Identifying Best Practices to Improve Evaluation and Management of In-Hospital Stroke: A Scientific Statement From the American Heart Association	US	American Heart Association	2022	https://www.ahajournals.org/doi/10.1161/STR.0000000000000402
Management of Stroke in Neonates and Children: A Scientific Statement From the American Heart Association/American Stroke Association	US	American Heart Association	2019	https://www.ahajournals.org/doi/10.1161/STR.0000000000000183
Management of Stroke Rehabilitation	US	American Heart Association	2019	https://www.healthquality.va.gov/guidelines/Rehab/stroke/VADoDStrokeRehabCPGFinal8292019.pdf
Perioperative Neurological Evaluation and Management to Lower the Risk of Acute Stroke in Patients Undergoing Noncardiac, Nonneurological Surgery: A Scientific Statement From the American Heart Association/American Stroke Association	US	American Heart Association	2021	https://www.ahajournals.org/doi/10.1161/CIR.0000000000000968
Primary Care of Adult Patients After Stroke: A Scientific Statement From the American Heart Association/American Stroke Association	US	American Heart Association	2022	https://www.ahajournals.org/doi/10.1161/STR.0000000000000382
Society of Interventional Radiology Training Guidelines for Endovascular Stroke Treatment	US	Society of Interventional Radiology	2019	https://www.jvir.org/article/S1051-0443(19)30706-7/fulltext
Stroke Prevention in Symptomatic Large Artery Intracranial Atherosclerosis Practice Advisory	US	American Academy of Neurology	2022	https://n.neurology.org/content/98/12/486
